# Determination of multidrug-resistant populations and molecular characterization of complex *Klebsiella* spp. in wild animals by multilocus sequence typing

**DOI:** 10.14202/vetworld.2022.1691-1698

**Published:** 2022-07-19

**Authors:** Alessandra Tammy Hayakawa Ito de Sousa, Marco Túlio dos Santos Costa, Stefhano Luis Cândido, Herica Makino, Thais Oliveira Morgado, Lucas Avelino Dandolini Pavelegini, Edson Moleta Colodel, Luciano Nakazato, Valéria Dutra

**Affiliations:** 1 Microbiology Laboratory of the Veterinary Hospital, Universidade Federal de Mato Grosso, Cuiabá, Mato Grosso, Brazil; 2 Center for Medicine and Research of Wild Animals, Universidade Federal de Mato Grosso, Cuiabá, Mato Grosso, Brazil; 3 Pathology Laboratory of the Veterinary Hospital, Universidade Federal de Mato Grosso, Cuiabá, Mato Grosso, Brazil

**Keywords:** antimicrobial, *Klebsiella*, multidrug resistance, multilocus sequence typing, susceptibility, wild animals

## Abstract

**Background and Aim:**

One of the most significant public health concerns is multidrug-resistant (MDR) microorganisms. *Klebsiella* spp. have been at the forefront of causing different types of infections such as bacteremia, urinary tract infections, pneumonia, enteritis, and sepsis in humans as well as animals. This study aimed to determine the genomic similarity between *Klebsiella* spp. isolated from wild animal samples and those described in the Institut Pasteur genomic database to verify the spread of resistant clones regionally in the state of Mato Grosso, and to compare the epidemiological data in different regions of Brazil and the world.

**Materials and Methods:**

Isolates from various sites of injury in wild animals were identified by sequencing the *16S rRNA* gene. Antimicrobial susceptibility testing was performed using the disk diffusion method to verify the resistance profile, and then, multilocus sequence typing was performed to verify the population structure and compare the isolates from other regions of Brazil and the world.

**Results:**

Twenty-three sequence types (STs) were observed; of these, 11 were new STs, as new alleles were detected. There was no predominant ST among the isolates. All isolates were MDR, with high rates of resistance to sulfonamides, ampicillin, amoxicillin, and nitrofurantoin and low resistance to meropenem, imipenem, and amikacin.

**Conclusion:**

Improving our understanding of the population structure of *Klebsiella* spp. in wild animals may help determine the source of infection during outbreaks in humans or animals, as the One Health concept emphasizes the interlinks between humans, animals, and environmental health.

## Introduction

The One Health concept recognizes that human, animal, and environmental health are interconnected, and one sector can affect the health of others. In addition to these interlinks, antimicrobial resistance is one of the biggest public health concerns worldwide, which adds to the crisis as resistant organisms are difficult to control [1].

*Klebsiella* spp. belong to the Enterobacteriaceae family and are Gram-negative, non-motile and facultative aerobic bacteria. *Klebsiella* can be found in the environment, from sources such as fresh and saltwater, sewage, soil, and vegetation. In addition, it is a part of the intestinal microbiota of humans and animals and is found in several species of domestic as well as wild animals, including mammals, fish, birds, mollusks, insects, and earthworms [2]. In a previous study in Brazil, with 697 isolates from domestic animals, the major clinical disorders observed were that of the urinary, enteric, mammary, reproductive, and respiratory systems, showing opportunistic behavior of the pathogen. The pathogenicity of *Klebsiella* spp. may have been attributed to a set of virulence factors and resistance genes [3].

*Klebsiella* spp. can become multidrug-resistant (MDR) due to the presence of resistance genes. This is a significant problem in human hospitals [4]. The genus *Klebsiella* has a high population diversity and is divided into seven phylogroups: Kp1 *Klebsiella pneumoniae* (sensu stricto), Kp2 *Klebsiella quasipneumoniae* subsp. *quasipneumoniae*, Kp3 *Klebsiella pneumoniae* subsp. *similipneumoniae*, Kp4 *Klebsiella variicola*, Kp5 *Klebsiella variicola* subsp. *tropicalensis*, Kp6 *Klebsiella quasivariicola*, and Kp7 *Klebsiella africanensis* [5–10]. However, phylogenetic studies of the population diversity have shown that *K. pneumoniae* is closely related to *K. quasipneumoniae* (subsp. *quasipneumoniae* and subsp. *similipneumoniae*) and *K. variicola* [8, 11, 12]. These have very similar biochemical characteristics; hence, differentiation by biochemical tests and partial sequencing of the 16S rRNA gene is not effective. Therefore, phylogenetic analysis of the *rpoB* gene is indicated for such differentiation [13].

The data available on infection in wild animals in the state of Mato Grosso, Brazil, are limited. These are divided into three biomes and considering the importance of *Klebsiella* spp., this study aimed to verify the population structure of resistant clones in this environment and compared it with the populations isolated from other regions of Brazil and the world. In this study, the isolates used were from wild animals, including free-living and captive wild birds, mammals, and reptiles, that inhabit different ecosystems, such as the Amazon, Cerrado, and Pantanal of the state of Mato Grosso, Brazil.

## Materials and Methods

### Ethical approval

The project was approved by the Ethics and Research Committee for the Use of Animals (CEUA) under 215 protocol number 23108.236834/2017-13.

### Study period and location

This study was carried out from January 2016 to December 2017. Wild animals were treated at the Department of Clinic and Surgery of Wild Animals of the Veterinary Hospital and also at the Center for Medicine and Research of Wild Animals of the Federal University of Mato Grosso (Cuiabá, Mato Grosso, Brazil) for medical consultation and evaluation. Samples were received and processed at the Laboratory of Veterinary Microbiology and Molecular Biology.

### Isolates

The 25 samples that were collected had been isolated from different lesion sites (Table-1). The isolates were seeded on 8% sheep blood agar (Sigma-Aldrich, Darmstadt, Germany) and MacConkey agar (Neogen Corporation, São Paulo, Brazil) at 37°C under aerobic conditions and characterized morphologically and biochemically as described by Quinn *et al*. [14].

**Table 1:** *Klebsiella* isolates with species, host, isolation site, ST, and CC of the 25 isolates.

Species	Host (scientific name)	Host (popular name)	Sites	ST	CC
*Klebsiella pneumoniae*	*Ramphastos toco*	Toco Toucan	Oral swab	4526	2654
*Klebsiella pneumoniae*	*Buteogallus meridionalis*	Caboclo Hawk	Air bag swab	3440	101
*Klebsiella pneumoniae*	*Ara ararauna*	Blue and yellow Macaw	Flood	35	35
*Klebsiella pneumoniae*	*Ara ararauna*	Blue and yellow Macaw	Lung	70	70
*Klebsiella pneumoniae*	*Ara ararauna*	Blue and yellow Macaw	Airbag swab	70	70
*Klebsiella pneumoniae*	*Platycercus eximius*	Eastern Rosella	Lung	4536	147
*Klebsiella pneumoniae*	*Anodorhynchus hyacinthinus*	Blue Macaw	Case	76	76
*Klebsiella pneumoniae*	*Anodorhynchus hyacinthinus*	Blue Macaw	Brain	4537	340
*Klebsiella pneumoniae*	*Anodorhynchus hyacinthinus*	Blue Macaw	Liver	4538	340
*Klebsiella pneumoniae*	*Anodorhynchus hyacinthinus*	Blue Macaw	Hind limb injury	4539	340
*Klebsiella pneumoniae*	*Cairina moschata*	Wild duck	Oral swab	4544	4544
*Klebsiella pneumoniae*	*Caiman yacare*	Alligator	Heart	107	107
*Klebsiella pneumoniae*	*Boa constrictor*	Boa constrictor	Skin lesion swab	1661	1661
*Klebsiella pneumoniae*	*Puma concolor*	Puma	Urine	1948	35
*Klebsiella pneumoniae*	*Sapajus apella*	Capuchin monkey	Rectal swab	1480	37
*Klebsiella pneumoniae*	*Cerdocyon thous*	Crab-eating fox	Oral swab	2855	2855
*Klebsiella pneumoniae*	*Tapirus terrestris*	Lowland tapirs	Rectal swab	107	107
*Klebsiella pneumoniae*	*Amazona aestiva*	Amazon parrot	Lung	3636	*Singletons*
*Klebsiella pneumoniae*	*Nasua nasua*	Brown-nosed coati	Rectal swab	4542	*Singletons*
*Klebsiella quasipneumoniae* subs *similipneumoniae*	*Anodorhynchus hyacinthinus*	Blue Macaw	Hind limb injury	3506	*Singletons*
*Klebsiella quasipneumoniae* subs *similipneumoniae*	*Nyctibius griseus*	Common potoo	Air bag swab	4541	*Singletons*
*Klebsiella quasipneumoniae* subs *similipneumoniae*	*Tigrisoma lineatum*	Socó	Cloacal swab	4543	*Singletons*
*Klebsiella quasipneumoniae* subs *similipneumoniae*	*Myrmecophaga tridactyla*	Anteater	Swab nasal	4677	*Singletons*
*Klebsiella quasipneumoniae* subs *quasipneumoniae*	*Cerdocyon thous*	Crab-eating fox	Rectal swab	3093	*Singletons*
*Klebsiella quasipneumoniae* subs *quasipneumoniae*	*Nasua nasua*	Brown-nosed coati	Rectal swab	4540	*Singletons*

ST=Sequence type, CC=Clonal complex. *Featured new STs

### Molecular identification

#### Deoxyribonucleic acid (DNA) extraction

The extraction of genomic DNA from the isolates was performed by inoculating colonies in brain-heart infusion broth and incubated in a shaking incubator at 37°C, overnight and then centrifuged at 13.304× *g* for 5 min; the precipitate was resuspended in 1 mL of lysis buffer (100 mM NaCl, 25 mM EDTA, 100 mM Tris-HCl [pH 8.0], 0.5% sodium dodecyl sulfate, and 0.1 mg proteinase K) and the extraction of DNA was performed by the phenol-chloroform method according to Sambrook and Russell [15], 1:1 volume of phenol-chloroform were added and vortexed, after which the mixture was centrifuged at 13.304× *g* for 5 min, the supernatant was collected in a fresh Eppendorf and 0.3 mM sodium acetate was added. This Eppendorf was subjected to centrifugation at 13.304× *g* for 5 min. The supernatant was discarded and the pellet was washed with 1 mL of 70% ethanol and centrifuged at 13.304× *g* for 5 min. The pellet was dried after discarding the supernatant and the DNA was resuspended in 50 mL of ultrapure water and stored at −20°C.

#### 16S rRNA gene polymerase chain reaction (PCR)

The extracted DNA was subjected to 16S rRNA gene PCR analysis. The oligonucleotide sequences used were 27F: 5′-AGAGTTTGATCCTGGCTCAG-3′ [16] and 1492R: 5′-GGTTACCTTGTTACGACTT-3′ [17]; these sequences amplify a 1512 bp fragment. A 25 μL reaction volume was obtained by mixing 10 ng DNA, 20 pmol of each oligonucleotide, 0.2 mM dNTPs, 3 mM MgCl2, 1× PCR buffer, 1 U Taq DNA polymerase (Invitrogen, Carlsbad, California, USA), and ultrapure water.

The reactions were performed in a thermal cycler machine MyCyclerTM (BioRad) with initial denaturation for 5 min at 95°C; 35 cycles of denaturation for 45 s at 95°C; hybridization for 1 min at 52°C; and extension for 1 min and 30 s at 72°C; and final extension for 7 min at 72°C.

#### DNA sequencing

Subsequently, the PCR product was purified using an Illustra ExoProStar kit (GE Healthcare Life Sciences, Buckinghamshire, UK). It was then used in the sequencing reaction, together with BigDye Terminator Cycle Sequencing Ready Reaction mix in an ABI 3500 Genetic Analyzer (Applied Biosystems, Foster City, CA, USA). Finally, the sequences were compared and deposited in the GenBank database using BLAST on the NCBI server (www.ncbi.nlm.nih.gov/BLAST) using the accession number 4325327. The isolates that had a ≥ 97% similarity with *Klebsiella* spp. were selected. Subsequently, DECIPHER software v2.0 (http://www2.decipher.codes/index.html) was used for chimera observation and application in other techniques.

#### Multilocus sequence typing (MLST)

Seven oligonucleotides were used as described in the protocol on the Pasteur Institute website http://bigsdb.pasteur.fr/klebsiella/klebsiella.htmlto perform MLST [18]. The primers and fragment sizes are described in Annexure-1.

#### Analysis of MLST data

The combination allele was determined after sequencing the seven genes, and the sequence type (ST) and clonal complex (CC) were identified. In addition, sequences that were not available in the database (new STs) were deposited in the Pasteur Institute database (http://bigsdb.pasteur.fr/*Klebsiella/Klebsiella*.html).

The genotypes of the isolates were analyzed in goeBURST 1.2.1, and the STs were assigned to the same group (CC) if they shared 6–7 loci with at least one other ST. Concatenate sequence alignment analysis of MLST genes and nucleotide matrix identity were performed on Clustal Omega (www.ebi.ac.uk).

#### Antimicrobial susceptibility profiling

Antimicrobial susceptibility testing was performed by the agar disk diffusion method, as described by Bauer *et al*. [19]. Nine classes of antimicrobials (Oxoid, UK) were tested, namely, penicillins (ampicillin, amoxicillin, and amoxicillin with clavulanate acid), cephalosporins (cephalexin), carbapenems (imipenem and meropenem), aminoglycosides (amikacin and gentamicin), quinolones (ciprofloxacin), phenicols (chloramphenicol), tetracyclines (doxycycline), nitrofurans (nitrofurantoin), and sulfonamides (sulfonamides and sulfonamides with trimethoprim). Resistance results were classified according to the criteria described by the Clinical and Laboratory Standards Institute and the Brazilian Committee on Antimicrobial Susceptibility Testing [20–22].

The isolates were classified according to their resistance profiles as described by Magiorakos *et al*. [23] and defined as MDR when they were resistant to one or more agents in three or more categories of antimicrobials and a heatmap was generated for visualization.

## Results

The isolates were obtained from 13 different isolation sites; the most frequent samples were: Stool (20%; 5/25), oral cavity (16%; 4/25), and lungs (12%; 3/25) (Table-1). In total, there were 25 isolates of *Klebsiella* spp. from wild animals, of which 60% (15/25) were from wild birds, 32% (8/25) were from mammalian species, and 8% (2/25) from reptiles. Sixteen (12 birds, three mammals, and one reptile) of these isolates were from free-living animals and nine were from animals in captivity (Table-1).

After 16S *rRNA* gene sequencing analyses, 76% (19/25) of the isolates were classified as *K. pneumoniae*, 16% (4/25) as *K. quasipneumoniae* subsp. *similipneumoniae*, and 8% (2/25) *K. quasipneumoniae* subsp. *quasipneumoniae*.

**Figure-1:**
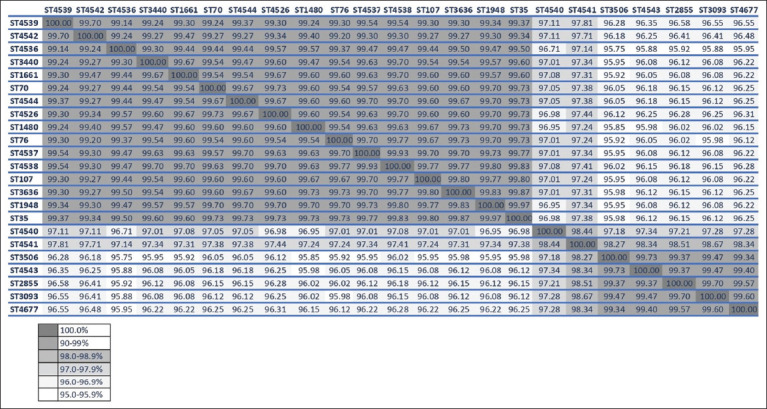
Percent identity and divergence score matrix of the seven concatenated gene nucleotide sequence type of *Klebsiella* spp. from wildlife animals.

Using the MLST technique, 23 STs were observed, 47.8% (11/23) of which were considered new (4526, 4536, 4537, 4538, 4539, 4540, 4541, 4542, 4543, 4544, and 4677). These STs were grouped into 11 CCs and eight singletons (Table-1). In concatenated sequences analysis, there were no indels or tetra-allelic single-nucleotide polymorphisms (SNPs). Matrix identity between isolates range has 96.58–100%. The number of polymorphic sites was 188 (6.24%) from 3012 nucleotide positions and diversity index (π) 0.01843 (Figure-1).

**Figure-2:**
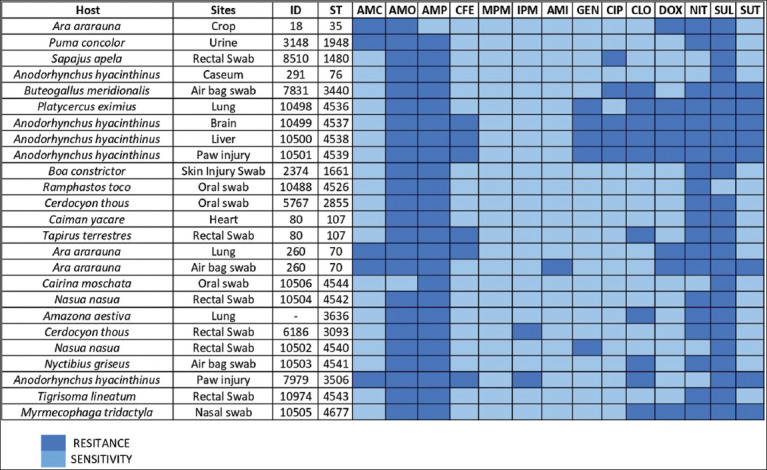
Heat map of antimicrobial resistance profile of complex *Klebsiella* spp. isolates by means of agar disk diffusion. AMO=Amoxicillin, AMC=Amoxicillin + clavulanic acid, AMP=Ampicillin, CFE=Cephalexin, MPM=Meropenem, IPM=Imipenem, GEN=Gentamicin, AMI=Amikacin, CIP=Ciprofloxacin, CLO=Chloramphenicol, DOX=Doxycycline, NIT=Nitrofurantoin, SUL=Sulfonamides, SUT=Sulfonamide+trimethoprim. *Samples classified as intermediate were regrouped with those classified as resistant.

All isolates were found to be MDR. The results showed the highest levels of resistance against sulfonamides 100% (25/25) and 96% (24/25) against ampicillin, amoxicillin, and nitrofurantoin. The antibiotics with the lowest resistance rates were meropenem (0%), imipenem (8%; 2/25), and amikacin (12%; 3/25) (Figure-2).

## Discussion

Three MDR species of *Klebsiella* were observed in this study, with a predominance of *K. pneumoniae*, mainly in birds, and the isolates presented a great clonal diversity. One of the biggest concerns in the public health community is the potential risk posed by the colonization of bacterial pathogens from wildlife and the consequent contamination of the environment, potential transfer to food crops, humans, and domestic animals. There is also a risk of dissemination of zoonotic diseases and transmission of MDR pathogens, which is one of the biggest health problems anticipated by the One Health concept [24].

There exist a few previous studies on genotyping of *Klebsiella* in wild animals [25–27], and in this study, new STs were found. The genotypes and CCs observed in these previous studies show occurrence both in human samples and in other domestic animals. In samples of beef, pork, chicken, and clinical human urine samples, 60 STs were described, and 21 (35%) were considered new, probably due to the low number of deposits in the database for *K. pneumoniae* isolates from animals [28]. In addition, *K. pneumoniae* isolates have high population diversity [2, 12, 29, 30], and new strains were discovered through this study with many polymorphic sites (SNP). There was no predominance of a strain and a variety of STs were found.

Two free-living blue and yellow Macaw isolates from our study were classified as ST70 belonging to CC70, which has already been identified in *K. pneumoniae* isolate from a human hospital in Rio de Janeiro, Brazil [31], and in India [32]; this ST70 is more associated with nosocomial infections in humans [33]. Other studies carried out with parrots and silver gulls (*Chroicocephalus novaehollandiae*) found several STs differing from our results [26].

In our study, primate isolate from a Capuchin monkey (*Sapajus apella*) was classified as ST1480, which belongs to CC37. This clonal group has been identified on inanimate surfaces in a hospital environment in Algeria [34]. The first described case, ST1480, was isolated in a human blood sample in New York in 2012 (id:8510 Gomez-Simmonds *et al*. [35]). In addition, there have been other reports of wild primates in captivity in Brazil, such as the golden-handed tamarin (*Saguinus midas midas*) and non-human primates (*Alouatta clamitans*) with different STs from the ones found in our study [25, 27].

Two isolates from our study belonged to CC35; these isolates were from animals in captivity. One ST35 isolate was from a blue and yellow Macaw, and the other ST1948 was from a puma. *K. pneumoniae* ST35 is an MDR clone and has been identified in humans from several countries [33, 36, 37]. It is also found in feces, milk from [id: 3084, id: 13421), broiler and turkey in Norway [38], urban rodents in China [39], and wastewater in Romania [40]. In addition, the ST1948 strain was described in blood isolate from a hospital in Taiwan and Laos [12, 41] and found in crows in Italy (id:13729).

We also found two isolates of *K. pneumoniae* ST107 in captive alligators and free-living tapirs. Furthermore, the same ST was observed in hospitals in China with several virulence factors and resistance genes, such as *K. pneumoniae* carbapenemase 2 *(bla-KPC2)* and New Delhi metallo-beta-lactamase 9 (*bla-NDM9*) [42, 43]. This ST107 has never been described before in animals; the first case of this strain was in the USA (id:80) and has been deposited on the website of Pasteur Institute.

There have been previous reports of *K. pneumoniae* ST76 infections worldwide. It has been identified in various cases such as a bird tracheal sample in the Netherlands [33], dogs in Italy, pigs in Thailand and Switzerland, and humans in countries such as Brazil, the United States, Italy, and Vietnam (Database of the Pasteur Institute) [12, 44], the same ST76 has been described in our study, in hyacinth macaws with caseous lesions.

The high rate of beta-lactam resistance, particularly ampicillin resistance, is due to the intrinsic resistance of *K. pneumoniae* (*SHV-1*) [12]. However, there was a low resistance to carbapenems. Notably, imipenem resistance was found in two free-living animals and was probably acquired from the environment in which these animals lived. We hypothesize that resistance to imipenem has been acquired from an environment contaminated with resistant pathogens and exchange of resistance genes since the use of carbapenems in animals is still limited to treating serious diseases in companion animals [29, 45]. As for the isolates of human origin, they have shown a high rate of resistance due to the presence of the KPC enzyme, as previously described [46].

Among the aminoglycosides, amikacin had a lower resistance rate than gentamicin. This may be due to the reduced presence of the methylase genes, *armA* and *rmtB*, which are the genes mainly involved in amikacin resistance in *Klebsiella* spp. [47]. Notably, this drug has been widely used to treat nosocomial infections [29, 46, 48].

The increase in MDR pathogens in wild, companion, and production animal species as well as in the environment has created a significant problem because of the transmission of these MDR pathogens between humans and animals [49, 50]. The proximity of human activities influences the emergence of antimicrobial resistance in wild animals, contributing to this worldwide problem in human and veterinary medicine [51].

In a study carried out on free-living porpoises (*Inia geoffrensis*) in the Amazon, Brazil, the resistance profile was analyzed to find resistant pathogens in these animals, with the aquatic environment acting as reservoirs of pathogenic bacteria, and these wild animals acting as the vectors, for transmission [51]. Monitoring and surveillance of antimicrobial resistance among aquatic life, bacteria, wildlife, domestic animals, humans, and the environment are essential to prevent the further emergence and spread of antimicrobial resistance [52].

The presence of antibiotic-resistant bacteria in wild animals may be evidence of the impact of human activities on natural ecosystems [24]. For example, wild birds can acquire these bacteria by ingesting food with these MDR pathogens through the fecal-oral route, and with this, they can act as sentinels of these MDR pathogens and spread to other places [52–54].

MDR *K. pneumoniae* isolates from animals are a major concern because they possess clinically relevant antimicrobial resistance genes; thus, these animals serve as reservoirs of the important determinants of resistance. This is worrisome as the previous study has shown that *Klebsiella* species are prone to nosocomial spread [55]. Therefore, it is advisable to avoid the spread of these high-risk clonal strains into the population [45]. This concern increases when it comes to wildlife environmental conservation.

## Conclusion

*Klebsiella* isolates from wild animals used in this study were MDR with a high rate of resistance to several antimicrobials. The population structure had great genetic diversity, and there was no predominance of one genotype. The presence of MDR *Klebsiella* in wild animals poses significant risks to public health, as these animals can act as reservoirs of MDR *K. pneumoniae* and *K. quasipneumoniae* strains and can be transferred to the environment and food through feces. Thorough knowledge of *Klebsiella* population structure is needed to determine the relationship between MDR organisms and the source of infection during outbreaks, providing data for greater control in public health and reducing the spread of resistant clones to avoid environmental propagation as observed in wild captive and free-living animals. Further research is needed to investigate the resistance genes and virulence factors to verify the pathogenicity of these isolates.

## Authors’ Contributions

ATHIS, LN, and VD: Conceptualization of the study, methodology, data analysis, interpretation of the data, and writing of the manuscript. MTSC, SLC, HM, LADP, EMC, LN, and VD: Investigation, sample collection, and laboratory work. TOM, EMC, LN, and VD: Supervision. All authors have read and approved the final manuscript.

## Annexure

**Annexure-1:** Oligonucleotides, sequences, and base pairs to be analyzed by polymerase chain reaction.

Gene	Sequence (5’ – 3’)	Product (pb)
*rpoB*	F-GGCGAAATGGCWGAGAACCA R-GAGTCTTCGAAGTTGTAACC	501
*gapA*	F-TGAAATATGACTCCACTCACGG R-CTTCAGAAGCGGCTTTGATGGCTT	450
*mdh*	F-CCCAACTCGCTTCAGGTTCAG R-CCGTTTTTCCCCAGCAGCAG	477
*pgi*	F-GAGAAAAACCTGCCTGTACTGCTGGC R-CGCGCCACGCTTTATAGCGGTTAAT	432
*phoE*	F-ACCTACCGCAACACCGACTTCTTCGG R-TGATCAGAACTGGTAGGTGAT	420
*infB*	F-CTCGCTGCTGGACTATATTCG R-CGCTTTCAGCTCAAGAACTTC	318
*tonB*	F-CTTTATACCTCGGTACATCAGGTT R-ATTCGCCGGCTGRGCRGAGAG	414
Sequencing *pgi*	F- CTGCTGGCGCTGATCGGCAT R- TTATAGCGGTTAATCAGGCCGT	420
Sequencing *infB*	F- ACTAAGGTTGCCTCCGGCGAAGC	318
